# Rheumatic Glossitis

**Published:** 1885-04

**Authors:** 


### Rheumatic Glossitis.

From a foreign exchange we learn that an alleged case of rheumatic glossitis is recorded by Dr. Carvallo in *La Cronica Medica.* The patient was a man aged forty, who one day found that his tongue began to enlarge and that articulation was difficult. The swelling increased so much that the tongue was no longer confined in the limits of the mouth. There was much difficulty in breathing; the lips were blue; the pulse was small; salivation was incessant; deglutition was painful; speech was impossible, and at times attacks of suffocation threatened dissolution. Leeches were applied to the angles of the lower jaw. Dr. Carvallo remembered to have read in M. Jaccoud's "Traite de Pathologie Interne," that Lawrence had written of the occurrence of glossitis as a consequence of rheumatism. The patient admitted that he had had rheumatism, and that he even then had rheumatic pains about the shoulders. An infusion of jaborandi was prescribed, and afterwards a draught containing salicylate of soda. The next day the tongue had diminished greatly in size, and had returned to the buccal cavity. The suggestion that such cases of swelling of the tongue may depend on a rheumatic agency is perhaps valuable; the proof that the case mentioned was of rheumatic nature cannot be said to have been given.

